# Stem Cells and Exosomes: New Therapies for Intervertebral Disc Degeneration

**DOI:** 10.3390/cells10092241

**Published:** 2021-08-29

**Authors:** Zoe Krut, Gadi Pelled, Dan Gazit, Zulma Gazit

**Affiliations:** 1 Department of Surgery, Cedars-Sinai Medical Center, Los Angeles, CA 90048, USA; zoe.krut@cshs.org (Z.K.); gadi.pelled@cshs.org (G.P.); dan.gazit@csmc.edu (D.G.); 2 Board of Governors Regenerative Medicine Institute, Cedars-Sinai Medical Center, Los Angeles, CA 90048, USA; 3 Department of Orthopedics, Cedars-Sinai Medical Center, Los Angeles, CA 90048, USA; 4 Faculty of Dental Medicine, Hebrew University of Jerusalem, Jerusalem 91120, Israel

**Keywords:** stem cells, exosomes, intervertebral disc, disc degeneration, disc regeneration

## Abstract

Intervertebral disc degeneration (IVDD) occurs as a result of an imbalance of the anabolic and catabolic processes in the intervertebral disc, leading to an alteration in the composition of the extracellular matrix (ECM), loss of nucleus pulposus (NP) cells, excessive oxidative stress and inflammation. Degeneration of the IVD occurs naturally with age, but mechanical trauma, lifestyle factors and certain genetic abnormalities can increase the likelihood of symptomatic disease progression. IVDD, often referred to as degenerative disc disease (DDD), poses an increasingly substantial financial burden due to the aging population and increasing incidence of obesity in the United States. Current treatments for IVDD include pharmacological and surgical interventions, but these lack the ability to stop the progression of disease and restore the functionality of the IVD. Biological therapies have been evaluated but show varying degrees of efficacy in reversing disc degeneration long-term. Stem cell-based therapies have shown promising results in the regeneration of the IVD, but face both biological and ethical limitations. Exosomes play an important role in intercellular communication, and stem cell-derived exosomes have been shown to maintain the therapeutic benefit of their origin cells without the associated risks. This review highlights the current state of research on the use of stem-cell derived exosomes in the treatment of IVDD.

## 1. Introduction

Intervertebral disc degeneration (IVDD) is the pathological condition associated with the degeneration of the intervertebral disc (IVD), an avascular structure composed of fibrous tissue and cartilage responsible for spinal kinematics and structural support [1]. IVDD is the most common cause of chronic low back pain (LBP), the leading cause of disability [2,3]. The prevalence of IVDD increases drastically with age; greater than 90% of people over the age of 50 present with degeneration of the intervertebral disc [4,5,6]. Other risk factors for IVDD include mechanical trauma, genetic predisposition, lifestyle factors and certain metabolic disorders [7,8,9]. Current treatments of IVDD aim to manage symptoms and minimize disability, but both pharmacological and surgical interventions can lead to complications, can be costly and have questionable efficacy [10]. Neither treatment option is capable of targeting the underlying cause of degeneration, and both lack the ability to hinder the progression of disease or reverse the degeneration to restore the precedent functionality of the disc [11].

Due to the limitations of the current treatments for IVDD, an increasing number of studies have focused on methods to regenerate the degenerative disc. IVDD is a multifactorial process, and treatments must be able to withstand the harsh microenvironment of the degenerative disc [12,13]. Various biological therapies, including the use of growth factors and platelet-rich plasma (PRP), have been evaluated in both preclinical and clinical studies, but many results have been underwhelming [14,15,16,17]. Preliminary preclinical studies have attempted to use CRISPR/Cas9 gene editing, or to improve current surgical methods using biomaterials, but studies of this kind are still in their infancy [18,19]. Stem cell-based therapies, both allogeneic and autologous, are an attractive method due to their ability to target many of the pathways that result in IVDD [20]. There has been progress with these studies, but there are concerns about a low survival rate of transplanted cells, which would make it difficult for researchers to control for cell viability and differentiation, and the risk of mutation [21,22]. With embryonic stem cells, there is also a potential lack of institutional readiness and societal acceptance [23].

Studies on the mechanism of stem cell-based therapies have provided increasing evidence that exosomes secreted by stem cells are responsible for the regenerative properties and efficacy in treating IVDD [24]. While initially considered a waste product, exosomes have recently been highlighted for their role in intercellular communication [25,26]. Exosomes show pronounced therapeutic competence for tissue regeneration through the maintenance of their endogenous stem cells, their ability to inhibit apoptosis and modulate the immune response, their enhancement of regenerative phenotypic traits, and stimulation of angiogenesis [27,28]. Stem cell-derived exosomes (SC-Exos), specifically, present a significant opportunity for the safe and effective treatment of IVDD because of their ability to maintain the therapeutic benefit of their origin cells without the risks associated with stem cell-based therapies [29]. This review highlights the current studies being conducted on stem cell-derived exosomes and their role in the treatment of degeneration of the intervertebral disc.

## 2. Intervertebral Disc Disease

### 2.1. Structure of the IVD

The IVD is comprised of the central nucleus pulposus (NP), surrounded by the annulus fibrosus (AF), and hyaline cartilaginous endplates which encompass these two structures at the junction of the vertebrae, known as the zygapophyseal joint. IVDs are responsible for one-third of the height of the spinal column, ranging from 7 to 10 mm thick depending on their location within the spine [30]. The IVD is an avascular structure and is therefore reliant on concentration gradients for the diffusion of nutrients and oxygen from the adjacent endplates [31]. Proteoglycans and type II collagen are the main components of the NP and aim to retain water to allow for the IVD to bear compressive loads. This is accomplished by the distribution of hydraulic pressure throughout the IVD [32]. The proteoglycans in the NP include both the larger aggrecan, which binds to hyaluronic acid, and several small leucine-rich proteoglycans. Aggrecan is largely responsible for the retention of water within the NP; a healthy NP is made of 66% to 86% water [33]. While of low density, approximately 5000/mm^3^, the NP contains cells that produce the components of the extracellular matrix (ECM) and help maintain the integrity of the NP; these cells are notochordal cells, which range from 25 to 85 µm in diameter, and NP cells, which are “chondrocyte-like” in nature and range from 17 to 23 µm in diameter [34]. NP cells primarily rely on glycolysis for metabolism and contain more mitochondria and rough endoplasmic reticulum (ER), packed with glycogen and cytoplasmic filaments, than notochordal cells [35,36]. While the role of notochordal cells in development is not fully understood, it is suggested that they are not only important for cell movement and proteoglycan synthesis of surrounding cells following senescence, but are also responsible for maintaining the gelatinous consistency of the NP because of their heightened ability to make proteoglycans [37,38].

The AF is stiffer than the NP and therefore able to provide more structural support. The AF is a ring-shaped disc of fibrous connective tissue which surrounds the NP. Type I collagen, the primary form of collagen in the AF, is arranged in concentric rings called lamellae, which are uniformly aligned in a parallel orientation and interconnected through translamellar bridges [39,40]. AF cells have been characterized as elongated fibroblasts with extended cytoplasmic processes of mesenchymal origin [41]. With aging, however, AF cells become more rounded and “chondrocyte-like” in nature [13,42].

The extracellular matrix (ECM) plays an essential role in the mechanical functionality of the IVD; the type I and type II collagen networks provide tensile strength, and water-binding proteoglycans, such as aggrecan, are vital for its integrity [43,44]. The ECM is similar in composition to other musculoskeletal connective tissues, containing collagen, proteoglycans, elastin and glycoproteins, but differs due to the fact that these components are commonly found in a fragmented form [45]. The ECM is a dynamic structure and proteinases synthesized by disc cells are responsible for the breakdown of these matrix macromolecules. Because of the avascular nature of the IVD, these fragmented components will naturally accumulate and degrade over time [46]. With degeneration, the most significant biochemical change to occur is the loss of proteoglycans, which results in the subsequent loss of glycosaminoglycans and the reduction in osmotic pressure of the disc matrix [9]. This has a major effect on the disc’s load-bearing behavior, which is evident by a degenerative disc’s tendency to bulge under pressure.

### 2.2. Pathogenesis of IVDD

IVDD is characterized by a painful degenerative disc caused by an imbalance of the anabolic and catabolic processes within the IVD. Metabolic dysregulation of NP cells results in a reduced ability to synthesize the components of the ECM, and an increase in the secretion of ECM degradative molecules [47,48]. As a result, the morphology of the disc will become more disorganized and the lamellae in the AF will become irregular [49]. Progressive IVDD involves the calcification of the tiny pores within the endplate, which results in impaired diffusion and a compromised exchange of gases and nutrients, furthering the degenerative cascade [17]. This imbalance leads to an alteration in the composition of the ECM, loss of NP cells, excessive oxidative stress, and inflammation, which ultimately results in a decreased capacity to bear compressive loads [50,51]. Dysregulation, combined with proteoglycan breakdown and a diminished water-binding capacity, will eventually lead to structural collapse.

Degeneration of the IVD is aggravated by inflammation, which plays an important role in the biological process and has emerged as a distinguishing factor in the appearance of the symptomatology of the diseased disc [12,52]. Intracellular reactive oxygen species (ROS) also play a role in the promotion of nucleotide-binding domain, leucine-rich-repeat containing family, pyrin domain-containing 3 (NLRP3) inflammasome activation and interleukin-1β release [53]. With metabolic dysregulation, advanced glycation end products (AGEs) accumulate in NP tissues and promote its degeneration, further increasing oxidative stress and the secretion of major inflammatory cytokines, upregulating ECM degradative enzymes and downregulating ECM structural components; thus, initiating a host immune response [54,55,56]. While the pathophysiology of IVDD has not been fully characterized, nerve infiltration is believed to be the source of the pain associated with the degeneration of the intervertebral disc [57].

## 3. Current Treatment of IVDD

Current treatments for disc degeneration range from changes in lifestyle to surgically invasive interventions. Nonpharmacological treatments such as exercise, weight loss and physical therapy are indicated in the treatment of IVDD, but only in cases where degeneration is not severe. Pharmacological treatments such as opiates, steroids and non-steroidal anti-inflammatory drugs (NSAIDs) are aimed at achieving pain control for better function and quality of life but are accompanied with their own risk of side effects and, for some, with the potential to develop dependence [10].

Operative interventions include discectomy with fusion and total disc replacement (TDR). Spinal fusion, also known as arthrodesis, is the process of fusing or joining two bones, and is currently considered the gold standard for the management of IVDD [58]. TDR, the replacement of the degenerative intervertebral disc with an artificial disc, is typically only indicated when there is single-level disc disease, and the facet joints are disease-free [59]. Despite the availability of surgical interventions, there is still a clear need for improved IVDD therapies that target the underlying causes of degeneration, rather than simply mitigate the associated symptoms. It is important that new interventions are not accompanied by a significant risk of complications or adverse side effects, like some current IVDD treatments. In hopes of targeting the root cause of disc degeneration, various biological and stem cell-based therapies have been examined.

## 4. Biological Therapies for IVDD

While IVDD is a multifactorial process, biomolecular therapies primarily focus their treatment efforts on the progressive decline in NP hydration due to the loss of proteoglycans and collagen, which results in a loss of mechanical tension in the AF collagen fibers and results in abnormal spinal axial loading forces and segmental instability [11]. Biomolecular therapies aim to target the diseased state, characterized by decreased anabolism and increased catabolism within the IVD, by introducing recombinant proteins or nucleic acids aiming to reverse the process of degeneration. The goal is to facilitate ECM synthesis and promote NP rehydration and nutrition. To do so, however, biological interventions must be able to mitigate the harsh, proinflammatory, pro-catabolic and anti-anabolic environment within the degenerative IVD.

When the investigation into the use of injectable biological therapies for the treatment of IVDD began, there was significant interest in conducting studies on the transforming growth factor (TGF) superfamily and, more specifically, bone morphogenetic proteins (BMPs). Unfortunately, studies on the use of BMPs, such as BMP-2, BMP-7, recombinant human connective tissue growth factor (rhCTGF) and TGF-β, in various animal models, have either shown minimal or no impact, or have reported serious adverse events (SAEs) [15,16,17,39,60,61]. A study conducted on human recombinant Growth/Differentiation Factor-5 (GDF-5) (rhGDF-5) showed that the stimulation of gene expression led to the synthesis of ECM proteins, type II collagen and aggrecan in IVD. As a result, a multicenter, randomized, double-blind, placebo-controlled clinical trial on the intradiscal injection of rhGDF-5 was conducted in 2014 to evaluate safety and tolerability (A Multicenter, Randomized, Double-blind, Placebo Controlled, Clinical Trial to Evaluate the Safety, Tolerability and Preliminary Effectiveness of 2 Doses of Intradiscal rhGDF-5 (Single Administration) for the Treatment of Early Stage Lumbar Disc Degeneration. Available online: https://clinicaltrials.gov/ct2/show/results/NCT01124006 (accessed on 2 July 2021)) [15]. The results of this study were never published, suggesting that treatment with rhGDF-5 either did not have a therapeutic effect or was accompanied by SAEs.

There is the risk that the activity of endogenous proteases within the IVD may degrade injected therapeutic proteins, meaning that the bioavailability and persistence of injected biomolecules may be transient, suggesting that doses may need to be high and repeated frequently [62,63]. With repeated intradiscal doses, however, there is the increasing risk of puncturing of the AF, and it has been previously established from patients who have undergone discography that injury to the AF accelerated disc degeneration [64]. Annular repair techniques, in both sheep and porcine models, are still being investigated and aim to work in conjunction with other treatment modalities [65].

Due to the mixed results of studies evaluating the efficacy of growth factors, studies on other therapies have been conducted [66,67,68]. A clinical trial on the intradiscal injection of a formulation including lactic acid, known as STA363, has been completed but the results have yet to be posted; it was hypothesized that the treatment of STA363 would result in the growth of fibrous, connective tissue in its 15 participants (A Single Ascending Dose Study of Safety and Tolerability of STA363 Compared to Placebo in 15 Patients with Chronic Discogenic Low Back Pain. Available online: https://clinicaltrials.gov/ct2/show/results/NCT03055845 (accessed on 8 July 2021)). A phase 1b, multicenter, double-blind, single ascending dose study is currently underway to evaluate the safety of intradiscal injection of AMG0103, a synthetic nuclear factor-κB decoy oligodeoxynucleotide, in patients with discogenic lumbar back pain (AMG0103 in Subjects with Chronic Discogenic Lumbar Back Pain. Available online: https://clinicaltrials.gov/ct2/show/NCT03263611 (accessed on 7 July 2021)). In addition, there have also been recent attempts to use CRISPR/Cas9 gene editing to repair dysfunctional gene regulation, but these approaches are still currently only being investigated in vitro [18]. With respect to therapeutic gene delivery, various vectors, including adenovirus, retrovirus and lentivirus, have been investigated, but safety remains a concern [69].

Platelet-rich plasma (PRP) is a fraction of plasma produced by centrifugal separation of whole blood and another potential treatment for disc degeneration [70]. A prospective, randomized controlled trial assessing intradiscal PRP injections in discogenic-mediated low back pain demonstrated improvements in pain and function in patients as early as eight weeks post-treatment [71]. While this singular clinical trial appeared to have somewhat promising results, there has been considerable inconsistency in pre-clinical animal models [14,72,73].

Non-coding RNAs, mainly microRNAs (miRNAs), long non-coding RNAs (IncRNAs) and circular RNAs (circRNAs), also play a role in the development of IVDD and can therefore be used as a potential treatment target. These post-transcriptional regulators are known to affect the expression of 30% of protein coding genes and numerous intracellular processes [51]. After incorporation into the RNA-induced silencing complex (RISC), miRNAs can target and inhibit the translation of multiple mRNAs, thereby regulating multiple intracellular processes including cell proliferation, apoptosis and cytokine release [74]. The overexpression of miR-410 has been shown to significantly inhibit pyroptosis by suppressing the NLRP3/caspase-1 pathway, presenting a potentially significant treatment target [56]. It has also been suggested that the development of nanoparticle technology could help with using miRNAs and circRNAs as therapies for IVDD [75].

Biological treatments have also been used to improve on the current surgical interventions for IVDD. Biomaterials used for tissue engineering scaffolds, including fibrin, silk, gelatin and PLGA, have been used in tandem to create biphasic scaffolds with the goal of engineering an artificial IVD which utilizes the unique structures and functions of both the NP and AF [76]. Implantation of injectable collagen hydrogels, such as Atelocollagen, showed the ability to prevent progression of IVD space narrowing and had viability and proliferative activity in an in vivo rabbit model, but clinical trials have not yet been conducted [19].

Currently there are no clinical trials utilizing tissue-engineered IVD (TE-IVD) transplantation, but a pilot study using a biomimetic protein polymer, which mimics the NP, uses NuCore^®^ injectable nucleus (Spine Wave, Inc., Shelton, CT, USA) was reported [77]. The use of biomaterials, while potentially useful on their own, appears to be greatly enhanced when stem cells are utilized in tandem [78,79]. As cell-based therapies are capable of targeting more than just one of the pathways that cause degeneration of the IVD, they may be more effective than other biological treatments currently being investigated.

## 5. Cell-Based Therapies for IVDD

Cell-based therapies could very well be the optimal treatment strategy for patients with mid-stage degeneration because they could directly address the decreased number of viable NP cells and stem cells within the diseased disc space. Mesenchymal stem cells (MSCs) have been the focus of most studies utilizing cell-based therapies for the treatment of IVDD, but other studies have evaluated the use of progenitor cells, hematopoietic stem cells, adipose-derived stem cells (ADSCs), discogenic cells and even fibroblasts. MSCs are multipotent, adult stem cells which can differentiate into osteocytes, adipocytes and chondrocytes, and are found in a variety of human tissues, including the bone marrow, adipose tissue, liver, intestine, connective muscle tissue, skin and umbilical cord [80]. The attractive attributes of MSCs include their ability to recruit into damaged sites and lower immunogenicity due to the lack of expression of any costimulatory molecules, thus suggesting that there is no requirement for the use of immunosuppressive agents with allogeneic transplantation [81]. MSCs also secrete cytokines, immune receptors and anti-inflammatory molecules, allowing them to regulate the microenvironment of the host tissue.

As the therapeutic potential of MSCs has already been established for various diseases, in various animal models, there have recently been several clinical trials investigating the intradiscal injection of autologous MSCs. A pilot phase I trial conducted in Spain utilized bone marrow derived MSCs (BMSCs) and showed that autologous BMSC transplantation is both feasible and safe. The analgesic effect of treatment with BMSCs approached 71% efficacy and was described as rapid because most improvement in pain (85%) was attained by 3 months. Unfortunately, this study did not show an improvement in disc height, but this is consistent with the results from spinal fusion, with or without TDR, and discectomy [20,82]. A phase I/II clinical trial designed to analyze the safety, feasibility and potential clinical efficacy of the implantation of autologous MSCs embedded in tricalcium phosphate, found that 80% of patients achieved lumbar fusion, and no adverse side effects related to the procedure were recorded [83]. There are currently clinical trials in the recruitment process, while others have been completed but have yet to publish their results (Efficacy of Intradiscal Injection of BM-MSC in Subjects with Chronic Low Back Pain (LBP) Due to Lumbar Degenerative Disc Disease (DDD) Unresponsive (RESPINE). Available online: https://clinicaltrials.gov/ct2/show/NCT03737461) (accessed on 8 July 2021), Safety and Preliminary Efficacy Study of Mesenchymal Precursor Cells (MPCs) in Subjects with Lumbar Back Pain. Available online: https://clinicaltrials.gov/ct2/show/NCT01290367 (accessed on 6 July 2021).

In addition to MSCs, other stem cells have been investigated for their potential to treat IVDD. Autologous hematopoietic stem cells have been of interest due to their differentiation and proliferative capacities, but the hypoxic environment of the degenerative IVD poses a challenge. A study utilizing hyperbaric oxygenation in 10 patients following percutaneous intradiscal injection of autologous hematopoietic precursor stem cells (HSCs) showed no improvement in discogenic pain after one year, and eight of 10 patients underwent surgical treatment within one year of study completion. This study suggested that the use of non-mesenchymal lineage cells in the treatment of IVDD may be limited [84]. In murine and rabbit models of chronic disc degeneration, adipose-derived stem cell (ADSC) transplantation promoted new expression of proteoglycans and increased levels of aggrecan, and exhibited elevated extracellular matrix anabolism and minimal ossification, but no clinical trials have directly utilized ADSCs for the treatment of IVDD [85,86]. ADSCs, however, have been implicated in a clinical trial which utilized stromal vascular fraction (SVF) obtained from a lipoaspirate procedure of fat tissue, in conjunction with PRP obtained from peripheral blood. The 15 participants in this clinical trial experienced improvements in flexion over the 6-month follow-up period and a reduction in pain and discomfort from baseline [87]. Notably, no SAEs were reported during the 12-month follow-up period. This study, however, lacked a placebo control, and therefore additional studies are necessary.

The use of autologous NP cells is another alternative to repair damaged or historically inflamed tissue by addressing multiple propagators of degeneration at once. A clinical trial on the use of autologous disc chondrocyte transplantation (ADCT) administered 12 weeks following discectomy, compared to discectomy alone to mitigate postoperative pain, showed that those who received ADCT had continual improvement of their pain, and that these results persisted at the two-year follow-up, but there was no difference in the mean IVD height between the experimental and control groups [88]. The ADCT system is currently available and utilized in Germany but has not been approved by the United States Food and Drug Administration (FDA) [89].

Autologous bone marrow aspirate and bone marrow concentrated cells (BMCs) contain multiple stem and progenitor cells, including MSCs, that can be autografted at the time of surgery. A clinical trial with 55 participants had bone marrow aspirate concentrate (BMAC) collected from the iliac crest and injected into the intradiscal space. There were significant improvements in the patients’ pain scores [90]. In addition, this study showed that patients who received >2000 colony-forming unit-fibroblasts (CFU-F) per milliliter of bone marrow aspirate had statistically significant improvements in pain scores compared to those who had less. There are two additional clinical trials which will further evaluate the intradiscal injection of BMAC, one that is currently recruiting participants and the other which is currently underway (A Prospective Study of Clinical Outcomes Following a Single Intradiscal Injection of Bone Marrow Aspirate Concentrate (BMAC) for Single Level Discogenic Low Back Pain. Available online: https://clinicaltrials.gov/ct2/show/NCT03912454 (accessed on 6 July 2021), Bone Marrow Concentrate (BMC) Injection in Intervertebral Discs. Available online: https://clinicaltrials.gov/ct2/show/NCT04559295 (accessed on 12 July 2021)). Allogeneic stem cells are also an attractive option due to their low harvesting costs, and because a primary harvesting operation is also not necessary in this case. A clinical trial completed in 2017 demonstrated the safety, feasibility and potential effect of allogeneic BMSCs in 24 participants with IVDD [11,22]. There is currently a clinical trial being analyzed that looked at the intradiscal injection of allogeneic mesenchymal precursor cells (MPCs) (Safety and Preliminary Efficacy Study of Mesenchymal Precursor Cells (MPCs) in Subjects with Lumbar Back Pain. Available online: https://clinicaltrials.gov/ct2/show/NCT01290367 (accessed on 8 July 2021)). Another potential treatment target is conditioned media (CM) from hMSCs, which was found to mitigate the effects of matrix metalloproteinases (MMPs), the enzymes responsible for ECM degradation, in a 3D in vitro disc cell pellet model [91].

A study in murine and rabbit IVDD models utilized discogenic cells from various human donors. In mice, discogenic cells were able to generate the components of the ECM. The discogenic cells persisted for 4 months after subcutaneous implantation and showed continual recruitment of proteoglycans and collagen. In rabbits, injection of allogeneic discogenic cells increased disc height by up to 15%. This study also demonstrated a lack of toxicity and tumorigenicity [92]. Human clinical testing of discogenic cells combined with a sodium hyaluronate carrier is ongoing in randomized controlled, double-blinded studies in the United States (Study to Evaluate the Safety and Preliminary Efficacy of IDCT, a Treatment for Symptomatic Lumbar Intervertebral Disc Degeneration. Available online: https://clinicaltrials.gov/ct2/show/NCT03347708 (accessed on 10 July 2021)) and in Japan (Study to Evaluate the Safety and Preliminary Efficacy of IDCT, a Treatment for Symptomatic Lumbar Disc Degeneration. Available online: https://clinicaltrials.gov/ct2/show/NCT03955315 (accessed on 10 July 2021)). A phase 2a study utilizing participants undergoing lumbar spinal fusion has completed evaluating the implantation of allogeneic osteoblastic cells with a ceramic scaffold, but the results have not yet been published (Phase 2a Study on Allogeneic Osteoblastic Cells Implantation in Lumbar Spinal Fusion. Available online: https://clinicaltrials.gov/ct2/show/NCT02205138 (accessed on 9 July 2021)). A recent publication on the use of dermal fibroblasts as a possible cellular transplant in Cynomolgus monkeys showed that injection of fibroblasts demonstrated retained IVD height, and it was hypothesized that this treatment resulted in reparative fibrosis repair [93]. This study was limited, however, because the fibroblast transplant occurred immediately at the time of disc injury, the treatment group was only followed for 8 weeks, and there was very broad variability in the MRI data.

Endogenous repair was another treatment target investigated in IVDD models, but the viability of tissue-specific progenitor cells, also known as endogenous progenitor cells, was significantly affected by the adverse microenvironment of the degenerative IVD (hypoxia, low pH, high mechanical load, inflammation, etc.) [94]. When the IVD has been seriously injured, the repair effect of progenitor cells on the IVD cannot meet the need for repair [95,96]. Moreover, progenitor cells have a decreased ability to respond to signals of injury when external pressure is exerted, as a high mechanical load can cause apoptosis in NP cells via a caspase-dependent mitochondrial pathway. A recent study successfully constructed a delivery system of endogenous progenitor cells based on pullulan microbeads and signaling molecules involved in cell recruitment into NP tissue, followed by the release of growth factors to complete the ECM remodeling, but there is significant research that still needs to be done before endogenous progenitor cells are used in the treatment of IVDD [97].

Nucleus pulposus-derived progenitor cells (NPPCs) did perform better than other progenitor cells in in vitro studies, as they have many similarities with MSCs in their surface markers, and have similar rates of cell proliferation and stem cell-like gene expression [98]. AF and cartilage endplate (CEP) progenitor cells also show specific similarities to BMSCs [99]. NPPCs are more adaptable to acidic microenvironments and actually undergo more proliferation and exhibit greater chondrogenic differentiation potential in hypoxic conditions. Progenitor cell homing, where endogenous cells are actively recruited into a desired anatomic site for therapeutic applications, when compared with exogenous cell transplantation, has better biological safety and rationality and would therefore be able to overcome some of the limitations that exogenous cell transplantation faces [95,96,100].

Despite the promising results outlined above, there are still several limitations that need to be addressed before the use of stem cell-based therapies can be introduced as a standard treatment in the clinical setting. A primary concern with stem cell-based therapies is with the potential loss of cells and diminished viability upon administration [28]. Due to the biological and ethical concerns that arise with the use of stem cells, it would be difficult to justify repeated implantation, which could potentially be necessary if challenges associated with administration cannot be adequately addressed [23]. Furthermore, the transplantation of exogenous cells is expensive. Even within clinical trials, there is currently no consensus with respect to the number of cells that ought to be transplanted; injections of BMSCs in clinical trials currently range from 6 × 10^6^ to 25 × 10^6^ cells (Treatment of Degenerative Disc Disease with Allogeneic Mesenchymal Stem Cells (MSV) (Disc_allo). Available online: https://clinicaltrials.gov/ct2/show/record/NCT01860417 (accessed on 7 July 2021), Safety and Preliminary Efficacy Study on Mesenchymal Precursor cells (MPCs) in Subjects with Lumbar Back Pain. Available online: https://clinicaltrials.gov/ct2/show/NCT01290367 (accessed on 9 July 2021)) [22]. Finally, while whole stem cells have been shown to impact IVD degeneration, a study aiming to establish the ability of MSCs to inhibit pyroptosis in NP cells found that when MSCs were treated with GW4869, which inhibits exosome secretion, the effect of the MSCs was abolished [24]. This led researchers to hypothesize that the effect of MSCs on pyroptosis is mainly caused by its derived exosomes, leading to the investigation into the use of stem cell-derived exosomes as a cell-free treatment for IVDD.

## 6. Stem Cell-Derived Exosomes as a Treatment for IVDD

### 6.1. Overview of Exosomes

Exosomes are nano-sized extracellular vesicles (EVs) composed of a lipid bilayer membrane, originating from multivesicular endosomes [101]. Exosomes are differentiated from the other EV subtypes—microvesicles (MVs) and apoptotic bodies—based on their biogenesis, physical composition, release pathways and function [102]. The diameter of an exosome ranges from 30–150 nm, with an average diameter of 100 nm, and exosomes have a density ranging from 1.13 g/mL to 1.19 g/mL [103,104]. Exosomes originate from the endocytic pathway, during which the inward budding of the intracellular endosomal membrane forms an early secretory endosome, intracellular multivesicular bodies (MVBs) containing intraluminal vesicles (ILVs) are formed, and the maturation of the endosome, as a result of acidification, causes ILVs to be secreted as exosomes by fusion with the plasma membrane [105].

Despite their identification in the late 1980s, exosomes were only recently recognized as novel mediators in intercellular communication [25]. Their tough lipid bilayer tolerates lyophilization and other extremes, while allowing for the retention of bioactivity, immune tolerability and efficiency after systemic delivery [29,106]. Secreted by various cell types and able to be isolated from small amounts of biological fluids, including blood, urine, semen, saliva, breast milk, bile and synovial fluid, exosomes impact cell–cell communication via transference of their various bioactive molecules: mRNA, microRNAs (miRNAs), proteins and bioactive lipids [26,107,108]. Proteomic analysis of exosomes has revealed more than 4000 different proteins, lending to their ability to engage in multifaceted functions [50,109]. Transferred exosomal mRNA can be translated after entering another cell; one study proposes that this type of RNA be called “exosomal shuttle RNA (esRNA)” [110].

Exosomes show pronounced therapeutic competence for tissue recovery through the maintenance of their endogenous stem cells, enhancement of regenerative phenotypic traits, inhibition of apoptosis with immune modulation and stimulation of angiogenesis [27,111]. The secretion of exosomes has been shown to be elevated in response to inflammation, hypoxia and acidic microenvironments, and can be used by tumors to induce immunosuppression and promote angiogenesis by delivering miRNA [112,113]. While the delivery of miRNA is contraindicated for patients with cancer, it may prove useful in the treatment of IVDD. Exosomes are suspected to restore damaged tissue and can uphold their therapeutic efficacy by transferring biologically active molecules and affecting target molecules, which regulate the gene expression and phenotype of damaged recipient cells [110,114]. In addition, exosomes are able to maintain viability under extreme conditions, and can even provide recipient cells with a resistance against oxidative stress [115,116].

### 6.2. Overview of Stem Cell-Derived Exosomes

Stem cell-derived exosomes (SC-Exos) have been used for the treatment of various forms of tissue injury in preclinical trials due to the maintenance of their stemness, induction of regenerative phenotypes, apoptosis inhibition and immune response regulation [117]. Wnt and mTOR pathways are master regulators required for MSC-Exos secretion, and they support the self-renewal of MSCs [118]. While the exact underlying mechanism of MSC-derived exosomes (MSC-Exos) in the restoration of damaged tissue has yet to be elucidated, research suggests that MSC-Exos are responsible for the maintenance of endogenous stem cells, induction of regenerative phenotypes by promoting cell proliferation and angiogenesis, protection of cells from apoptosis, the attenuation of oxidative stress and the adjustment of the immune response through the delivery of immunomodulatory mediators to damaged tissue [117,119].

### 6.3. Effect of Stem Cell-Derived Exosomes on IVDD

Various studies have evaluated the use of stem-cell derived exosomes in the treatment of IVDD (Table 1) [24,119,120,121,122]. Bone marrow-derived mesenchymal stem cell-derived exosomes (BMSC-Exos) were found to reduce interleukin-1β-induced inflammatory cytokine secretion and MAPK signaling activation by delivering miR142-3p, which targets mixed lineage kinase 3 (MLK3) [123]. Another study found that BMSC-Exos have an abundance of miR-532-5p, which targets RASSF5 [124]. The knockdown of RASSF5 is suspected to decrease apoptotic cells. An in vitro study on the efficacy of hBMSC-derived exosomes resulted in more than a 50% increase in cell proliferation and decreases in cellular apoptosis in 3D human degenerative disc cell cultures, and ECM production was observed as early as day 7 [125]. NP cell-derived exosomes can not only induce the differentiation of MSCs into NP-like cells in vitro, but were also found to promote the migration of MSCs and the downregulation of the Notch1 pathway [126,127]. NP cells from a rodent herniation model were found to produce exosomes containing miR223, which has been shown to downregulate inflammation through modulation of the NF-κB pathway [128,129,130]. The reduction in notochordal cell number with age is believed to be associated with the onset of IVD degeneration, and as a result, treatment with notochordal cell-derived exosomes was found to increase DNA and glycosaminoglycan content in human NP cell microaggregates, although the underlying mechanism was not analyzed [131,132].

In vivo studies on the use of stem cell-derived exosomes have more recently been conducted (Table 2). In a rabbit IVDD model, exosomes significantly prevented the progression of degenerative disease, confirming that the NLRP3 inflammasome is an effective target for disc degeneration, and that the injection of exosomes presents a promising therapeutic strategy [50,133]. In addition, it has been suggested that exosomes might supply mitochondrial proteins to NP cells and that damaged mitochondria in a degenerative disc could be restored [50]. MSC-Exos play an anti-pyroptosis role by suppressing the NLRP3 pathway, and it was proposed that this effect was attributed to miR-410, which, when derived from MSC-Exos, could directly bind to NLRP3mRNA [24,117]. In a rat model, intradiscal injection of BMSC-Exos alleviated NP cell apoptosis and IVD degeneration via miR-21 contained in the exosomes [134]. Exosomal miR-21 restrains the phosphatase and tensin homolog (PTEN), which results in the activation of the PI3K-AKT pathway [24]. MSC-Exos could also potentially decrease ER stress-induced apoptosis by activating AKT and ERK signaling. Delivery of BMSC-Exos in vivo modulated ER stress-related apoptosis and diminished the progression of degeneration in a rat tail model [135]. Human placental MSC (hPLMSC)-derived exosomes carrying AntagomiR-4450 were verified for their therapeutic effects on mouse NP cells in vivo and in vitro. Inhibiting miR-4450 upregulates ZNF121, which alleviates inflammation, apoptosis and damage to NP cells [136]. In vivo experiments in a rat model also indicate that sub-endplate injection of MSC-Exos can ameliorate IVDD, and it is suggested that this mechanism is related to the regulation of miR-31-5p and ATF6-related ER stress [137].

While the majority of in vivo studies have been conducted on MSC-Exos, exosomes secreted by other cell lines have also been evaluated for their potential use in tissue regeneration. A rat model was used to evaluate the intradiscal injection of exosomes derived from induced pluripotent stem cell (iPS)-derived MSCs (iMSCs), and this study showed that injection diminished the progression of IVDD and the senescence of NP cells, and was capable of restoring IVD height [138]. A study on normal cartilage endplate stem cell (CESC)-derived exosomes (N-Exos) showed a decrease in the apoptotic rate of NP cells. Utilizing a rat model of IVDD, this study showed that N-Exos attenuated the progression of disc degeneration by activation of the phosphatidylinositol 3-kinase (PI3K), AKT and autophagy pathways [139].

**Table 1:** Stem cell-derived exosomes for IVDD therapy referenced in this review.

Cell Type	Cell Origin	Scope of Study	Reference
Mesenchymal Stem Cells (MSCs)	Human	In vitro and in vivo	[24,137]
Bone Marrow-Derived Mesenchymal Stem Cells (BMSCs)	Rat	In vitro	[123]
	Mouse	In vitro	[124]
	Human	In vitro	[119,125,127]
	Human	In vitro and in vivo	[134,135]
Placental-Derived Mesenchymal Stem Cells (PLMSCs)	Human	In vitro and in vivo	[136]
Induced Pluripotent Stem Cell-Derived Mesenchymal Stem Cells (iMSCs)	Human	In vitro	[120]
	Human	In vitro and In vivo	[138]
Embryonic Stem Cell (ESC)-Derived Hematopoietic Stem Cells (HSCs)	Mouse	In vitro	[121]
Chondrocytes	Rabbit	In vitro	[122]
Fibroblasts	Human	In vitro and in vivo	[24]
Cartilage Endplate Stem Cells (CESCs)	Rat	In vitro and in vivo	[139]
Nucleus Pulposus (NP) Cells	Rat	In vitro	[126]
	Human	In vitro	[127]
Notochordal Cells	Pig	In vitro	[131]

**Table 2:** In vivo studies utilizing stem-cell derived exosomes for IVDD therapy.

Origin of Exosomes	Author	Experimental Objective	Animal Model	Results	Reference
MSCs	Zhang et al., 2020	To demonstrate NLRP3-mediated NP cell pyroptosis induction in IVDD mice model and identify regulators of this process	Mouse	Treatment with MSC-Exos and miR-410 reversed the increased protein levels of NLRP3 and alleviated the severity degree of IVDD	[24]
BMSCs	Xia et al., 2019	To investigate the therapeutic effect of exosomes via a reduction in NLRP3 inflammasome expression	Rabbit	Treatment with BMSC-Exos attenuates the progression of IVDD and delays matrix degradation	[50]
BMSCs	Cheng et al., 2018	To evaluate the protective effect of MSC-Exos on NP cell apoptosis and IVDD, and the regulatory effect of miRNAs	Rat	Intradiscal injection of MSC-Exos alleviates NP cell apoptosis via miR-21 contained in exosomes	[134]
BMSCs	Liao et al., 2019	To determine if the delivery of MSC-Exos could modulate endoplasmic reticulum (ER) stress in the IVD	Rat	Delivery of MSC-Exos modulates ER stress-related apoptosis in AGEs-associated IVDD	[135]
PLMSCs	Yuan et al., 2020	To elucidate the potential therapeutic role of human placental MSC-derived exosomes carrying AntagomiR-4450	Mouse	Inhibition of miR-4450 alleviates inflammation, apoptosis and damage to NP cells by upregulating ZNF121	[136]
MSCs	Xie et al., 2020	To characterize the effect and mechanism of MSC-derived exosomes and the inhibition of apoptosis and calcification in endplate chondrocytes (EPCs)	Rat	Sub-endplate injection of MSC-Exos reduces apoptosis and calcification in EPCs via regulation of miR-31-5p and ATF6-related ER stress	[137]
iMSCs	Sun et al., 2021	To explore the therapeutic effect of exosomes derived from induced pluripotent stem cell (iPS)-derived MSCs (iMSCs) on IVDD	Rat	Intradiscal injection could rejuvenate senescent NP cells and restore IVD height 4 weeks after needle puncture	[138]
CESCs	Luo et al., 2021	To compare the use of normal and degenerated cartilage endplate stem cell-derived exosomes to diminish apoptosis of NP cells	Rat	CEP inflammation aggravates IVDD and normal CESC-derived exosomes are better equipped to decrease the apoptotic rate of NP cells by activating the PI3K, AKT and autophagy pathways	[139]

## 7. Discussion

**Figure 1:**
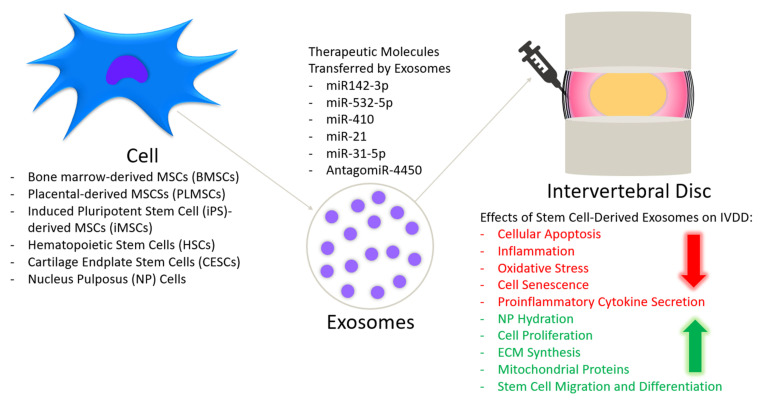
Stem cell-derived exosomes for IVDD treatment.

In this review, we summarized the current state of research on biological therapies for IVDD and highlighted new studies focused on stem cell-derived exosomes (Figure 1). Exosomes have the potential to be a more beneficial treatment than stem cell-based therapies because the therapeutic benefit of their origin cells is maintained, despite not being a cell, and therefore they do not have the associated risks of immunoreactivity, differentiation to the an undesired adult cell lineage, tumorigenesis or unwanted gene mutation [29]. There is also the potential to genetically modify exosomes to express special ligands, such as chemokine receptors, that will better direct them toward the site of injury and transfer small molecular drugs directly to the target sites, enabling exosomes to act as a targeted drug delivery mechanism [140,141,142]. While the avascular nature of the IVD poses a threat to the efficacy of systemically injected MSC-Exos, endeavors to prolong their half-life may enable this to be become a more prominent method of delivery [143,144]. The potential low yield of MSC-Exos, compared to MSCs, could be overcome by technical advancements in the expansion of MSCs and the enhanced release of exosomes [144]. It has been suggested that culturing MSCs in a low-density culture environment can activate exosome-mediated cell signaling and promote exosomes secretion [145]. It might be beneficial to consider the age, passage and differentiation state of the parental stem cells, as this can impact exosomes secretion, differentiation potential and the exosomal cargo transferred from MSCs [146,147]. Viability is also not a concern with exosomes, as it is with stem cell-based therapies. One study suggested that exosomes are potentially much easier to use post-thaw, and there is preliminary evidence that the thawing process may alter exosomal membranes so that they are absorbed more easily by target cells, although more research is needed to confirm this finding [148].

Currently there is only one clinical trial relating to the treatment of IVDD using exosomes, set to be conducted in India once all participants have been recruited. This trial is focused on the intradiscal injection of PRP that has been enriched with exosomes (Intra-discal Injection of Platelet-rich Plasma (PRP) Enriched with Exosomes in Chronic Low Back Pain. Available online: https://clinicaltrials.gov/ct2/show/NCT04849429 (accessed on 2 July 2021)). While stem cell-derived exosomes are a promising treatment for IVDD, there are many challenges that need to be addressed before they can be brought into the clinical setting. These include creating a method of selecting suitable donors to produce MSCs for the production of exosomes, the development of an exosome isolation protocol that meets good manufacturing practice (GMP) standards and a consensus on the required criteria and markers that need to be present before exosomes can be delivered to patients. There is also an immense need for methods to evaluate and ensure the safety of this patient population, which will need to be addressed and implemented prior to the initiation of clinical trials. It will be necessary to establish the course of action should the patient experience off-target or adverse side effects. 

Standardization will need to begin with the selection of parental stem cells and will require effective upscaling of the processes which ensure that the specific culture environment, age, passage and differentiation state are uniform. Currently, isolation and purification techniques for exosomes are not uniform, affecting the potential reproducibility of the current research; the isolation of exosomes from raw biological fluids can be challenging because other components, including microvesicles, may overlap in size, highlighting the need for standardization of these processes [149,150]. There is also the need for standardization of the release criterion of exosomes, which includes the size, surface marker expression and cargo. It will be vital to evaluate how exosome isolation techniques and the parental stem cell culture environment, which has been found to impact the contents and functions of their secreted exosomes, specifically impacts the efficacy of stem cell-derived exosomes in IVDD [117,151]. In the future, it will be necessary to develop assays capable of predicting the therapeutic potency of MSC-Exos that have high clinical sensitivity and specificity [152]. Because of the complexity of the role MSC-Exos in various diseases, it will most likely be necessary to develop assays that are disease specific [153].

As is the case with MSCs, the optimal dose of MSC-Exos is unknown, and even the best route of administration is still unclear, requiring further research [154,155]. Due to the avascular nature of the IVD and the limitations observed with the systemic injection of MSCs, it is suspected that direct injection of MSC-Exos will be the most effective delivery method for the treatment of IVDD [22]; however, an in vivo study published in 2020 administered MSC-Exos via injection into the tail vein, rather than the intradiscal injection method noted in other studies [24]. While the current in vivo studies only utilized a single dose, if systemic injections were to be implemented multiple doses may be necessary to achieve and maintain a therapeutic effect. In this case, it would be necessary to determine the safety and efficacy of multiple infusions, as well as the required dose and frequency. Finally, there are also concerns regarding legal classification, institutional readiness, and infrastructural support for successful clinical translation [121]. Although exosomes are not cells, their origination from stem cells may pose a challenge in terms of defining their legal classification and receiving approval from the U.S. FDA for use in the clinical setting [115]. Once these considerations are addressed, clinical trials on the use of stem cell-derived exosomes for the treatment of IVDD will be able to commence.

## Data Availability

Not applicable.
